# Assessing Baveno VI Criteria Using Liver Stiffness Measured with a 2D-Shear Wave Elastography Technique

**DOI:** 10.3390/diagnostics11050737

**Published:** 2021-04-21

**Authors:** Renata Fofiu, Felix Bende, Alina Popescu, Roxana Șirli, Bogdan Miuţescu, Ioan Sporea

**Affiliations:** Department of Gastroenterology and Hepatology, “Victor Babeș” University of Medicine and Pharmacy, 300041 Timișoara, Romania; renata.fofiu@yahoo.com (R.F.); alinamircea.popescu@gmail.com (A.P.); roxanasirli@gmail.com (R.Ș.); bmiutescu@yahoo.com (B.M.); isporea@umft.ro (I.S.)

**Keywords:** Baveno criteria, portal hypertension, high risk varices, 2D-SWE

## Abstract

The present study evaluates the performance of Baveno VI criteria, using liver stiffness (LS) assessed with a 2D-SWE elastography technique, for predicting high-risk varices (HRV) in patients with compensated advanced chronic liver disease (cACLD). A secondary aim was to determine whether the use of spleen stiffness measurements (SSMs), as additional criteria, increases the performance of the 2D-SWE Baveno VI criteria. Data were collected from 208 subjects with cACLD, who underwent abdominal ultrasound, liver and spleen stiffness measurements, and upper digestive endoscopy. HRV were defined as grade 1 esophageal varices (EV) with red wale marks, grade 2/3 EV, and gastric varices. A total of 35.6% (74/208) of the included subjects had HRV. The optimal LS cut-off value for predicting HRV was 12 kPa (AUROC-0.80). Using both LS cut-off value < 12 kPa and a platelet cut-off value > 150 × 109 cells/L as criteria to exclude HRV, 52/208 (25%) subjects were selected, 88.5% (46/52) were without EV, 9.6% (5/52) had grade 1 EV, and 1.9% (1/52) had HRV. Thus 98% of the subjects were correctly classified as having or not having HRV and 25% of the surveillance endoscopies could have been avoided. Using SS < 13.2 kPa and a platelet cut-off value > 150 × 109 cells/L as additional criteria for the patients that were outside the initial ones, 32.7% of the surveillance endoscopies could have been avoided.

## 1. Introduction

The evaluation of portal hypertension (PH) in a non-invasive manner is an increasingly used approach and steps are being taken to identify new, easily reproducible, and widely available non-invasive markers.

Assessing the status of PH with early and correct identification of patients at high risk of developing complications associated with PH, such as ascites, esophageal varices (EV), and even variceal bleeding is of the utmost importance [1]. 

The gold standard method to ascertain the presence and significance of PH is the invasive measurement of the hepatic venous pressure gradient (HVPG), while upper digestive endoscopy is the gold standard method for diagnosing esophageal and gastric varices. HVPG is normally within the 1–5 mmHg range and becomes clinically significant when it reaches values of 10 mmHg or above [2]. Both techniques are invasive and more difficult for patients to accept and therefore non-invasive, widely available, and easily reproducible techniques are needed, especially in patients with newly diagnosed compensated advanced chronic liver disease (cACLD), when screening with non-invasive markers is preferable in order to define the best timing to perform endoscopy or other invasive techniques [3].

Several non-invasive markers have been validated as useful for predicting the presence of EV and one of the most widely used approaches to correctly identify patients with cACLD who can avoid upper digestive endoscopy is the use of Baveno VI criteria. Baveno VI consensus suggests that the combination of liver stiffness (LS) < 20 kPa by transient elastography (TE) and platelet count > 150 × 109 cells/L could avoid endoscopy in cALCD, as the possibility of having high-risk varices (HRV) is very low when these criteria are fulfilled, being less than 5% [4].

Both LS and platelet count are non-invasive markers which have been exhaustively studied and validated for predicting the presence and severity of EV. LS is one of the most studied and confirmed non-invasive predictors of clinically significant portal hypertension (CSPH) and HRV. Several studies evaluated LS measurements by TE and concluded that LS-TE is an easily reproducible non-invasive parameter that correlates with HVPG and the presence of EV [5,6,7]. A very good correlation between LS measurements performed by 2D Shear Wave Elastography (2D-SWE) techniques and the presence of CSPH has also been demonstrated in published studies. Unlike TE, 2D-SWE techniques have the advantage of being integrated into an ultrasound system that allows, in addition to elastography evaluation, also B-mode, Doppler, or contrast-enhanced evaluation [8,9,10,11,12].

When it comes to combining several parameters for HRV prediction, the platelet count is frequently used along with other well-known parameters. Regarding the performance of platelet values for predicting the presence and severity of EV, the results are controversial. Even if some studies demonstrated a good performance of platelet count alone to predict the presence and severity of EV, most studies have shown that their performance is better when used along with other non-invasive markers [4,13,14,15,16].

Another non-invasive parameter that is useful for predicting the presence and severity of EV is spleen stiffness measurement (SSM). Studies have found a clear and reproducible correlation between SSM and the presence and severity of PH [10,11,17,18,19]. 

The present study aims to evaluate the performance of Baveno VI criteria, using LS assessed with a 2D-SWE technique for predicting the presence of high-risk varices in patients with cACLD. A secondary aim was to determine whether the use of SSM as an additional criterion increases the performance of the previously established criteria. 

## 2. Materials and Methods 

### 2.1. Subjects

**Figure 1:**
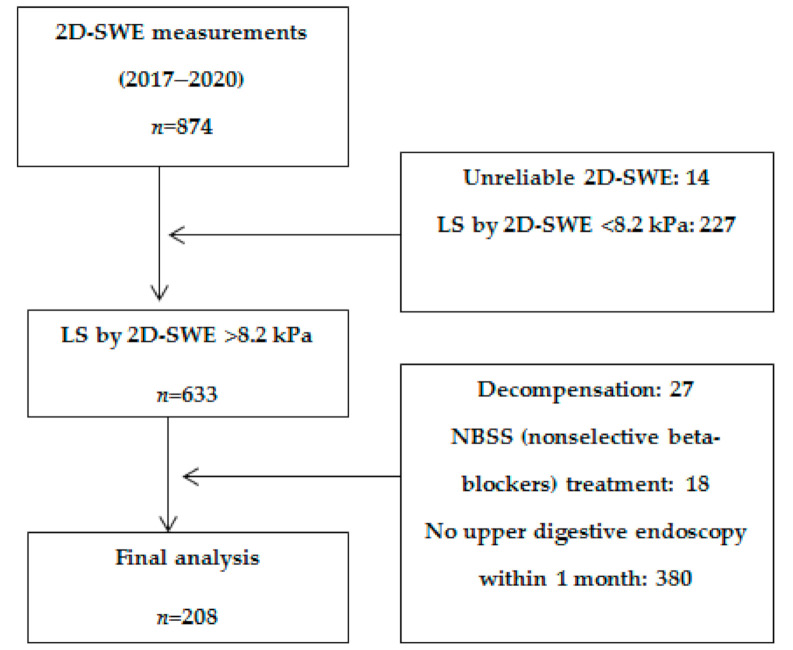
Flow chart of patient enrolment.

Data were retrospectively collected from 874 subjects who underwent LS measurements by means of 2D-SWE from General Electric between January 2017 and January 2020 in a tertiary gastroenterology center. After applying the exclusion criteria, 208 subjects were included in the final analysis (Figure 1).

Inclusion criteria for all subjects were: the ability to provide informed consent, age ≥ 18 years old, patients with cACLD based on clinical, biological, and elastography (LS by 2D-SWE > 8.2 kPa) criteria, and patients in whom upper digestive endoscopy was performed within 1 month of the elastography evaluation [20].

Exclusion criteria were: LS by 2D-SWE ≤ 8.2 kPa, unreliable LSM by 2D-SWE, decompensated patients or patients known to have a decompensation episode in the past, patients with focal liver lesions or with non-cirrhotic PH, and patients in whom upper digestive endoscopy was not performed within one month of the elastography evaluation.

All the included subjects underwent complete clinical evaluation, upper digestive endoscopy, and LSM by 2D-SWE technique from General Electric (LOGIQ E9, GE Healthcare, Wauwatosa, WI, USA), generally in the same admission, but not at more than 1-month intervals. The following data were collected: age, gender, body mass index (BMI), etiology of liver disease, laboratory findings, and LS values. A subgroup of 124 subjects underwent SSM by 2D-SWE technique from General Electric, also within a 1-month interval from the endoscopic evaluation. A previously established cut-off value was used for SS when the SS criteria were established [21].

All the participants signed informed consent for performing elastography measurements and upper digestive endoscopy. The study was approved by the Institutional Ethics Committee of County Emergency Clinical Hospital “Pius Brînzeu”, Timișoara (No 17/16.02.2019) and was performed following the last revised version of the Helsinki Declaration.

### 2.2. 2D-SWE Evaluation

LS and SS evaluation by 2D-SWE was performed using a LOGIQ E9 system (GE Healthcare, Wauwatosa, WI, USA) (version 2.0). All measurements were performed with the patient in fasting conditions (at least 6 h) in a supine position with the arm in maximum abduction, by intercostal approach, in the right liver lobe and the splenic parenchyma, respectively, in the best acquired acoustic window [22,23].

The measurements were performed using a C1-6-D convex probe and the SWE region-of-interest (ROI) was placed at least 1–2 cm below the liver capsule, in a region free of large vessels for LSM, while for SSM, mostly in the superior pole of the spleen. The patient was asked to suspend breathing when the most appropriate area of parenchyma was identified, and afterward image acquisition was initiated. A total of 2–3 colored SWE image frames were recorded for 5 s, acquiring a total of 10 SWE frames. The ROI was placed within each SWE frame and the stiffness inside the ROI was calculated.

The system automatically calculates the median value and IQR of the valid measurements. Reliable measurements were defined as the median value of 10 measurements acquired in a homogenous area, with an IQR/M < 0.30. Strictly 10 consecutive measurements were performed and measurements were considered failures if no value was obtained after 10 attempts.

2D-SWE measurements were performed by two experienced operators, blinded to the endoscopy results. 

### 2.3. Upper Digestive Endoscopy

All subjects underwent upper digestive endoscopy performed by an experienced endoscopist (more than 5 years of diagnostic endoscopy), who was blinded to the elastography measurements, within 1 month of 2D-SWE evaluation. The presence and the grade of EV, as well as the presence of gastric varices or portal hypertensive gastropathy, were recorded. EV that were described as grade 1 with red wale marks or medium/large (grade 2/3) EV were defined as HRV. Any type of gastric varices were also defined as HRV.

### 2.4. Statistical Analysis

The statistical analysis was performed using MedCalc Statistical Software version 12.5.0.0 (MedCalc Software, Ostend, Belgium) and SPSS Statistics for Windows, Version 17.0. (SPSS Inc., Chicago, IL, USA). The Kolmogorov–Smirnov test was used to test the normality of distribution. The mean value and standard deviation were calculated for numerical variables with normal distribution, while in cases of non-normal distribution, median values and range intervals were used, whereas categorical variables were reported as the number (proportion) of patients with/without the specific characteristic. Student’s *t*-test was used for group comparisons of continuous variables with a normal distribution and a Mann–Whitney U-test was applied for variables with non-normal distribution. Group comparisons of categorical variables were performed using Pearson’s χ^2^-test. Results were reported as odds ratios (ORs) and 95% confidence intervals (CIs) whenever appropriate.

Univariate regression analysis and multivariate regression analysis were used in order to identify the factors associated with the presence of HRV. The coefficients obtained from the logistic regression were expressed in terms of odds ratio with 95% confidence intervals. 

Areas under receiver operating characteristic (AUROC) curves were calculated for 2D-SWE values to identify the optimal cut-off values for LS, platelet count, and the two parameters combined. The optimal cut-off values were calculated from AUROC curve analysis, by using the Bayesian analysis, using the optimal criterion (the cut-off value with the highest sum of Se and Sp) and avoiding the misclassification of true positive subjects. Positive predictive value (PPV—true positive cases/all positive cases), negative predictive value (NPV—true negative cases/all negative cases) and diagnostic accuracy (sum of true positive and true negative cases/total number of cases) were calculated. Ninety-five percent confidence intervals (95% CI) were calculated for each predictive test and a *p*-value < 0.05 was considered to reveal statistical significance. The performance of combined non-invasive tests for predicting HRVs was assessed by the number and percentage of spared endoscopies and misclassified HRV rates.

## 3. Results

Our study population consisted of 208 participants, 39.4% (82/208) subjects without EV and 60.6% (126/208) subjects with EV. A total of 58.7% (74/126) of the subjects with EV had HRV. The main characteristics of the subjects included are summarized in Table 1. 

**Figure 2:**
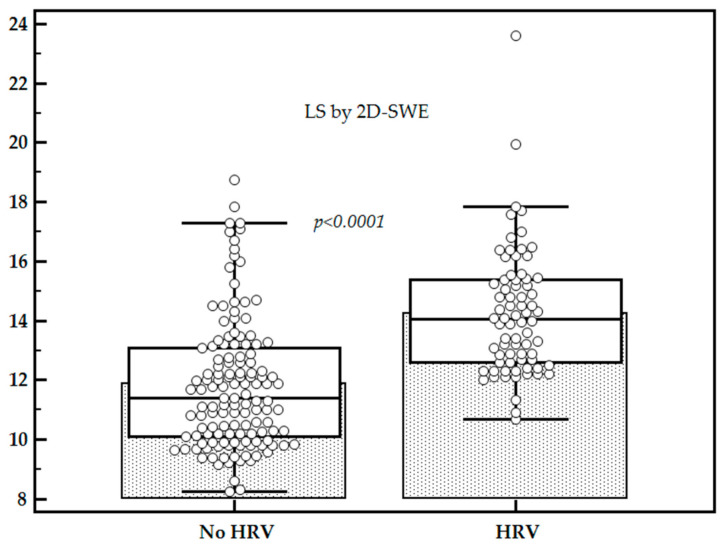
Comparison between liver stiffness (LS) values in subjects with high-risk varices (HRV) vs. subjects without HRV.

**Figure 3:**
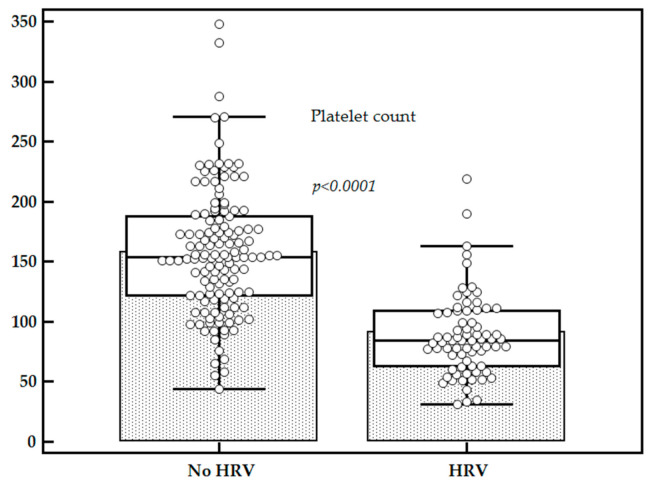
Comparison between platelets count in subjects with HRV vs. subjects without HRV.

Mean LS values were significantly higher for patients with HRV as compared to those without (14.25 ± 2.12 kPa vs. 11.89 ± 2.39 kPa, *p* < 0.0001), while the mean platelet count (×10^9^/L) was significantly lower for patients with HRV as compared to those without (91.33 ± 43.38 vs. 158.19 ± 52.15, *p* < 0.0001) (Figure 2 and Figure 3).

The association between LS measurements by 2D-SWE and the presence of HRV, as well as the association between platelet count and HRV, was tested in univariate regression analysis and both LS values by TE (*p* < 0.001) and platelet count (*p* < 0.001) were independent predictors for the presence of HRV. In multivariate logistic regression the model including LS measurements and platelets count showed that both parameters were associated with the presence of HRV: LSMs (β = 0.052 ± 0.01, *p* < 0.0001, OR = 1.4 [1.17–1.68]) and platelet count (β = −0.0036 ± 0.00048, *p* < 0.0001, OR = 0.97 [0.96–0.98]).

The optimal LS cut-off value (with the higher sum of sensitivity and specificity) for predicting the presence of HRV was 12 kPa (AUROC-0.80, Se-94.5%, Sp-60.5%, PPV-56.9%, NPV-95.3%). A total of 40.8% (85/208) of the subjects had LS by 2D-SWE.GE < 12 kPa and 30.6% (26/85) of them had EV of which 15.3% (4/26) were HRV. Furthermore, 59.2% (123/208) of the subjects had LS by 2D-SWE.GE > 12 kPa and 81.3% (100/123) of them had EV, of which 70% (70/100) were HRV (Table 2).

A cut-off value of 150 × 10^9^ cells/L was used for platelets (AUROC-0.87, Se-93.2%, Sp-58.9%, PPV-55.6%, NPV-94%). A total of 40.4% (84/208) of the subjects had platelets >150 × 10^9^ cells/L and 23.8% (20/84) of them had EV, of which 25% (5/20) were HRV. Furthermore, 59.6% (124/208) of the included subjects had platelets < 150 × 10^9^ cells/L and 85.5% (106/124) of them had any grade EV of which 65% (69/106) were HRV (Table 2).

**Figure 4:**
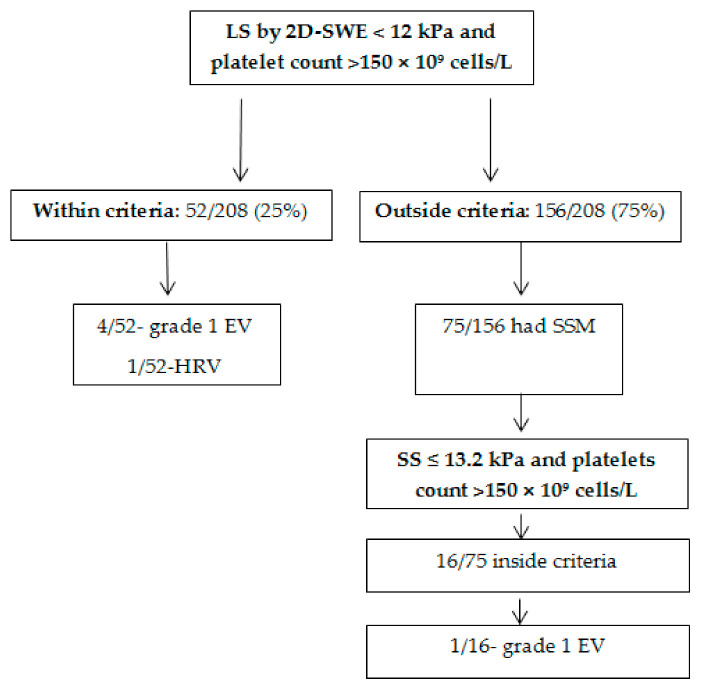
Flow chart of HRV detection.

The 2D-SWE Baveno criteria were applied and 25% (52/208) of the subjects met both criteria (LS by 2D-SWE < 12 kPa and platelets count > 150 × 10^9^ cells/L). In this group, 9.6% (5/52) of subjects had EV, with only one of them having HRV. A total of 75% (156/208) of the included subjects were classified as being outside the 2D-SWE Baveno criteria with 77% (120/156) of them having any grade EV, of which 60.8% (73/120) were HRV (Table 2, Figure 4).

Using both LS by 2D-SWE and platelets count criteria to detect HRV yielded 98.6% [92.7–100.0] sensitivity, 38.0% [29.8–46.8] specificity, 46.8% [38.8–54.9] PPV, 98.1% [89.6–100.0] NPV, 1.59 [1.4–1.8] LR+, 0.036 [0.005–0.3] LR− (Table 3). In total, 98% of the subjects were correctly classified as having or not having HRV and only one patient with HRV was misclassified. By applying these criteria we could have potentially avoided 25% of surveillance endoscopies in our study group.

A total of 75/156 subjects that were found to be outside the established criteria (2D-SWE Baveno criteria) had SS measurements performed with 2D-SWE. In order to classify the subjects that did not meet the initial 2D-SWE Baveno VI criteria, a previously established SS cut-off value for predicting HRV together with the same cut-off value for platelets count was used as additional criteria in this subgroup of subjects (SS criteria: (SS ≤ 13.2 kPa and platelet count > 150 × 10^9^ cells/L).

SS ≤ 13.2 kPa had 87.8% Se, 68.3% Sp, 65.5% PPV, 89.1% NPV, with 0.84 AUROC to predict HRV, while platelets count > 150 × 10^9^ cells/L had 93.2% Se, 58.9% Sp, 55.6% PPV, 94% NPV, and an AUROC of 0.87. A total of 16/75 (21.3%) subjects were inside the SS criteria. None of the subjects that met the SS criteria had HRV and a single subject had grade I EV (Figure 4). Adding these subjects to those who met the initial criteria (2D-SWE Baveno VI), 32.7% of the surveillance endoscopies could have been avoided.

## 4. Discussion

Considering the importance of assessing the status of PH in all patients with cACLD at the time of diagnosis, several non-invasive markers and different multi-parametric approaches were studied. Conclusions regarding the superiority of using a step-by-step algorithm or a multi-parametric approach are inconsistent among studies, but better performances were observed in the multi-parametric approaches as compared to their individual use [2,18,21,24]. In the early stages of cACLD, intrahepatic factors (increased vascular resistance due to hepatic fibrosis) correlate well with the portal pressure, but the development of EV and, even more, of HRV, is also affected by extrahepatic factors. Therefore, the association of LS with extrahepatic factors linked with portal hypertension (platelet count) improves the performance of LS in predicting the presence of HRV [21,24,25,26].

The Baveno VI criteria (platelet count > 150 × 10^9^ cells/L and LSM by TE < 20 kPa) or the new Expanded-Baveno VI criteria (platelet count > 110 × 10^9^ cells/L and LSM by TE < 25 kPa) are the most used and validated approaches to correctly identify patients with cACLD who can avoid upper digestive endoscopy [27].

Given the fact that each elastography technique has its own performance regarding the non-invasive evaluation of PH, but also to overcome the disadvantages of TE, this study aimed to evaluate the Baveno criteria using a 2D-SWE technique for the assessment of LS for predicting HRV in patients with cACLD. 

Both good feasibility and intra- and inter-observer reproducibility proven by previous studies [20,28], as well as the fact that there are a limited number of studies on the performance of this method for predicting the presence and severity of EV, led us to test the Baveno criteria using the 2D-SWE technique from General Electric in our cohort. A total of 98% of the subjects that met our newly established Baveno criteria were correctly classified as having or not having HRV and only one patient with HRV was misclassified. 

By applying these criteria we could have avoided 25% of surveillance endoscopies, results that are quite similar to those obtained with the original Baveno VI criteria (21% spared endoscopies) [28]. Avoiding endoscopy, when not needed, contributes both to improving the life quality of patients with cACLD and reducing costs.

Higher mean LS values were found in patients with HRV as compared to those without, while mean platelet count values were significantly lower for patients with HRV, in accordance with other published studies [8,21,29].

The optimal LS cut-off value for predicting HRV in our study group was 12 kPa, with an AUC of 0.80 (sensitivity 94.5%, specificity 60.5%, PPV 56.9%, NPV % 95.3%), results that are quite similar to those retrieved in other studies [12]. Stefanescu et al. compared the performance of LS assessed with 2D.SWE.GE vs. TE to predict CSPH and concluded that the diagnostic performance is good in the subgroup of compensated patients, not being inferior to TE [12]. However, the post-test probability of diagnosing CSPH was 17%, higher using 2D-SWE than TE, suggesting its better feasibility. It was also concluded that 2D-SWE.GE seems to be easier to perform and less time-consuming, with five measurements being enough for an accurate estimation of LS [12].

Given the non-inferiority of 2D-SWE as compared to TE and the fact that other studies, although few, unanimously agree that LS evaluated with 2D-SWE has a good performance for the detection of CSPH and HRV, we consider that the approach of our study is scientifically supported [12,21].

A very similar approach to ours was has been proposed by Garcovich et al. [30], who performed a study on 195 subjects and tested the Baveno VI criteria using LS evaluated with a point shear wave elastography technique implemented on the iU22 ultrasound system (Philips Healthcare, Bothell, WA, USA)–ElastPQ. Coincidentally, although the elastography technique is different, the same cut-off value of LS (12 kPa) for predicting HRV was calculated. Using this cut-off value (LS < 12 kPa) and the cut-off value of >150 × 10^9^ cells/L for platelets count as criteria, 75/195 subjects were selected as being inside the “BAVElastPQ” criteria. Similar to the results of our study, only one patient with HRV was misclassified using the established criteria. The advantage of this study is that a high percentage of patients (38%) met the criteria, so a significant number of endoscopies could have been avoided (38% of the whole cohort). 

In our study only 25% (52/208) of the subjects met the initial criteria, so it can be stated that 25% of the endoscopies performed could have been avoided. In order to increase this number, we applied SS criteria to the subjects who did not meet the initial criteria and in whom SS evaluation was available. Thus, 16 subjects met the new criteria and all were correctly classified as having or not having HRV. Adding these subjects to those who met the initial criteria, we obtained a rate of 32.7% of endoscopies that could have been avoided.

A relevant limitation of the study is its retrospective nature, but since the interval between the elastography evaluation, upper digestive endoscopy, and laboratory findings does not exceed one month, this limitation is partially overcome. Furthermore, an important limitation is the fact that SSMs were not available in all patients, but considering the retrospective nature of the study, this could not be helped. Another limitation is related to the distribution of subjects according to etiology as 73% of the included subjects were cACLD patients with HCV infection. Furthermore, the inclusion of a larger number of patients in the study group, as well as the validation of our results by an independent group, either internal or external, would increase the value of the study. Taking into account these aspects, further studies performed on cohorts with a larger number of patients with a more homogenous distribution of etiologies are necessary.

In conclusion, Baveno VI criteria, using LS assessed with a 2D-SWE elastography technique (instead of TE) indicated good performance for HRV prediction in cACLD subjects, with a satisfactory rate of spared endoscopies. SS measurements can increase the performance of 2D-SWE Baveno VI criteria to predict HRV.

## Figures and Tables

**Table 1:** Main characteristics of the included subjects.

Parameter	All Subjects (*n* = 208)	no HRV *n* = 134 (64.4%)	HRV *n* = 74 (35.6%)	*p* Value
Age (years)	58.2 ± 10.4	58.7 ± 10.3	57.2 ± 10.7	*p* = 0.322
Gender				
Female	58.2% (121/208)	68% (91)	40.5% (30)	*p* = 0.0002
Male	41.8% (87/208)	32% (43)	59.5% (44)	*p* = 0.0002
Etiology				
HCV	73% (152/208)	82% (110)	56.8% (42)	*p* = 0.0002
HBV	5.8% (12/208)	4.5% (6)	8.1% (6)	*p* = 0.450
ALD	16.9% (35/208)	10.5% (14)	28.4% (21/74)	*p* = 0.0019
NAFLD	2.4% (5/208)	1.5% (2)	4% (3/74)	*p* = 0.511
PBC	1.9% (4/208)	1.5% (2)	2.7% (2/74)	*p* = 0.939
Platelet count (×10^9^/L)	134.40 ± 58.66	158.19 ± 52.15	91.33 ± 43.38	*p* < 0.0001
LS by 2D-SWE (kPa)	12.70 ± 2.56	11.89 ± 2.39	14.25 ± 2.12	*p* < 0.0001

Data are presented as number and percentage or mean ± standard deviation; *n*—number; HCV—hepatitis C virus; HBV—hepatitis B virus; ALD—Alcohol-related liver disease; NAFLD—non-alcoholic fatty liver disease; PBC—Primary biliary cirrhosis; LS—liver stiffness; HRV—high-risk varices.

**Table 2:** Distribution of subjects according to EV.

	Without EV	No HRV (Gr 1 EV)	HRV
All subjects	82/208 (39.4%)	52/208 (25%)	74/208 (35.6%)
LS < 12 kPa (*n* = 85)	59/85 (69.4%)	22/85 (25.9%)	4/85 (4.7%)
LS > 12 kPa (*n* = 123)	23/123 (18.7%)	30/123 (24.4%)	70/123 (56.9%)
Platelets > 150 000/mm^3^ (*n* = 84)	64/84 (76.2%)	15/84 (17.9%)	5/84 (5.9%)
Platelets < 150 000/mm^3^ (*n* = 124)	18/124 (14.5%)	37/124 (29.8%)	69/124 (55.7%)
Within 2D-SWE Baveno criteria (*n* = 52)	47/52 (90.4%)	4/52 (7.7%)	1/52 (1.9%)
Outside 2D-SWE Baveno criteria (*n* = 156)	36/156 (23.1%)	47/156 (30.1%)	73/156 (46.8%)

2D-SWE Baveno criteria are: LS by 2D-SWE < 12 kPa and platelets count > 150 × 10^9^ cells/L; LS—liver stiffness; HRV—high-risk varices; EV—esophageal varices; *n*-number.

**Table 3:** Diagnostic performance for each variable in the prediction of HRV.

Parameter	LS by 2D-SWE	Platelet Count	2D-SWE Baveno Criteria
Se (%) (95% CI)	94.5 [86.7–98.5]	93.2 [84.9–97.8]	98.6 [92.7–100.0]
Sp (%) (95% CI)	60.5 [51.6–68.8]	58.9 [50.1–67.4]	38.0 [29.8–46.8]
PPV (%) (95% CI)	56.9 [47.7–65.8]	55.6 [46.5–64.6]	46.8 [38.8–54.9]
NPV (%) (95% CI)	95.3 [88.3–98.7]	94.0 [86.7–98.0]	98.1 [89.6–100.0]
LR+ (95% CI)	2.4 [1.9–3.0]	2.2 [1.8–2.8]	1. 6 [1.4–1.8]
LR− (95% CI)	0.08 [0.03–0.2]	0.11 [0.05–0.3]	0.036 [0.005–0.3]

2D-SWE Baveno criteria are: LS by 2D-SWE < 12 kPa and platelets count > 150 × 10^9^ cells/L; LS—liver stiffness.

## Data Availability

The data underlying the findings of the study are available on request to the corresponding author (e-mail address: bendefelix@gmail.com).
